# Hipertensão Arterial Pulmonar com Envolvimento Venoso Associado à Esclerose Sistêmica – uma Nova Variante Patogênica no Gene ABCC8

**DOI:** 10.36660/abc.20250175

**Published:** 2025-12-11

**Authors:** Daniel Inácio Cazeiro, João Cravo, Mariana Pereira Silva, João R. Inácio, Oana Moldovan, Catarina Sousa, Catarina Lopes Resende, João Eurico Fonseca, Fausto J. Pinto, Rui Plácido

**Affiliations:** 1 University of Lisbon Unidade Local de Saúde de Santa Maria Department of Cardiology Lisbon Portugal Department of Cardiology, Unidade Local de Saúde de Santa Maria, CAML, CCUL@RISE, Faculty of Medicine, University of Lisbon, Lisbon – Portugal; 2 University of Lisbon Faculty of Medicine Department of Rheumatology and Metabolic Bone Diseases Lisbon Portugal Department of Rheumatology and Metabolic Bone Diseases, Unidade Local de Saúde de Santa Maria, CAML, Faculty of Medicine, University of Lisbon, Lisbon – Portugal; 3 University of Lisbon Faculty of Medicine Department of Radiology Lisbon Portugal Department of Radiology, Unidade Local de Saúde de Santa Maria, CAML, CCUL@RISE, Faculty of Medicine, University of Lisbon, Lisbon – Portugal; 4 University of Lisbon Faculty of Medicine Department of Medical Genetics Lisbon Portugal Department of Medical Genetics, Unidade Local de Saúde de Santa Maria, CAML, CCUL@RISE, Faculty of Medicine, University of Lisbon, Lisbon – Portugal; 5 Faculdade de Medicina da Universidade de Lisboa Center for Disease Mechanisms Research Lisbon Portugal Center for Disease Mechanisms Research, Faculdade de Medicina da Universidade de Lisboa, Lisbon – Portugal

**Keywords:** Hipertensão Pulmonar, Genética, Terapêutica

Uma mulher de 48 anos, de origem guineense-bissauense, com hipertensão arterial sistêmica, se apresentou no Pronto-Socorro com histórico de 5 meses de piora da fadiga, dispneia e edema periférico. Trabalhava como faxineira, sem exposição ocupacional e sem histórico familiar de hipertensão arterial pulmonar (HAP). Na admissão, apresentava hipoxemia, estertores crepitantes bibasais à ausculta e edema nos membros inferiores. O exame físico revelou telangiectasias palmares.

A investigação inicial revelou hipoxemia e níveis elevados de NT-proBNP. O eletrocardiograma mostrou desvio do eixo para a direita e sinais de hipertrofia ventricular direita. A angiotomografia computadorizada do tórax (angioTC) excluiu embolia pulmonar e revelou opacidades bilaterais em vidro fosco, dilatação das câmaras cardíacas direitas e da artéria pulmonar (AP) – Figura 1A – e dilatação esofágica difusa. O ecocardiograma transtorácico demonstrou ventrículo esquerdo em forma de D, dilatação das câmaras cardíacas direitas, insuficiência tricúspide grave e alta probabilidade ecocardiográfica de hipertensão pulmonar (HP).

**Figura 1:**
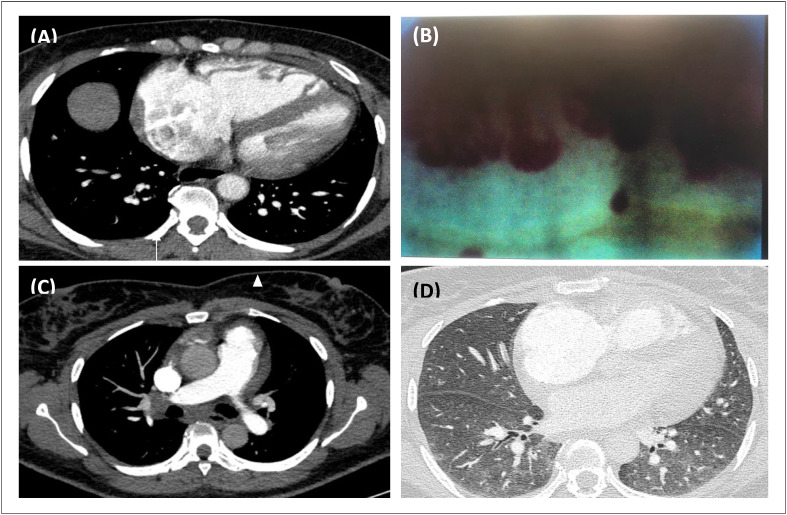
A) Capilaroscopia com padrão de esclerodermia ativa - perda moderada da densidade capilar, capilares gigantes (setas) e micro-hemorragias (ponta de seta) são evidentes. B) Angiotomografia computadorizada de tórax com contraste pulmonar demonstra dilatação do átrio e do ventrículo direitos, compatível com hipertensão pulmonar. C) Imagens axiais de tomografia computadorizada de tórax de acompanhamento mostrando linfadenopatia hilar e subcarinal bilateral. D) Imagens axiais pulmonares mostrando opacidades em vidro fosco bilaterais difusas nos lobos inferiores.

A paciente foi internada e iniciou oxigenoterapia (OT) e furosemida, com melhora clínica. Uma equipe multidisciplinar realizou investigação diagnóstica adicional para HP. A capilaroscopia revelou capilares gigantes e micro-hemorragias, compatíveis com esclerodermia ativa (Figura 1B). Os testes imunológicos detectaram altos títulos de anticorpos antinucleares e anticentrômero B. Os testes de função pulmonar (TFP) revelaram uma redução isolada e grave da capacidade de difusão de monóxido de carbono (DLCO) – 53% do previsto. As sorologias para HIV e hepatite B/C foram negativas. A ultrassonografia abdominal e a cintilografia de ventilação-perfusão não apresentaram alterações.

O cateterismo cardíaco direito confirmou HP pré-capilar, com teste de vasorreatividade negativo.

Um painel de sequenciamento de nova geração (NGS) de 16 genes envolvidos na fisiopatologia da HAP identificou uma variante heterozigótica patogênica no gene ABCC8 (NM_000352.6:c.4306C>T; p. Arg1436Te). O NGS foi realizado utilizando a tecnologia Twist Bioscience e um sequenciador NovaSeq 6000 (Illumina^®^). A análise de CNV foi realizada através do software DRAGEN (versão 07.021.572.3.6.3). Nenhuma outra variante foi identificada no painel. Um resumo das características e da localização da variante patogênica pode ser encontrado na Tabela 1 e na Figura 2, respectivamente.

**Tabela 1:** Resumo das características da variante patogênica ABCC8 identificada no paciente

Nomenclatura HGVS		ABCC8(NM_000352.6):c.4306C>T; p.(Arg1436Ter)
**Anotação cromossômica**		chr11:17395611C>T
**Localização**		11p15.1, exon 35, códon 1436
**Modo de herança**		Autossômico dominante/recessivo
**Zigosidade**		Heterozigoto (frequência alélica de 47%)
**Impacto da codificação**		Nonsense
**Classificação ACMG**		Patogênico (PVS1, PM2, PS4)
**Frequência populacional**		GnomAD Exomas: 0,00000358 Genomas GnomAD: 0,00000657
**Pontuações patogênicas:**		
	➢ CADD (escala Phred)	37
	➢ DANN	0,99
	➢ GERP++	>5,0
	➢ MutaçãoTaster 2025	Deletério
	➢ Previsão NMD	Positivo

Dados extraídos do ClinVar,^12^ VarSome,^13^ Bohnen et al.^14^ e Lago-Docampo et al.^15^

**Figura 2:**
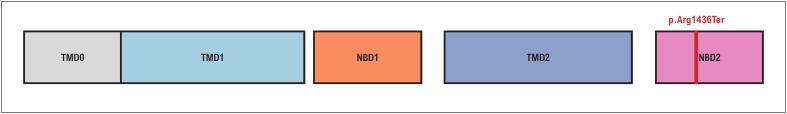
Localização estrutural da variante p. Arg1436Ter da ABCC8. Estrutura de domínio da proteína SUR1 (ABCC8) mostrando a localização da variante p. Arg1436Ter (linha vermelha). A variante trunca o segundo domínio de ligação a nucleotídeos (NBD2), podendo levar à perda de função da subunidade SUR1.

Esses achados confirmaram o diagnóstico de HAP associada à esclerose sistêmica (ES) - forma limitada. Foi iniciada terapia vasodilatadora com tadalafila e anbrisentana. O paciente recebeu alta no 27º dia de internação, mantendo o uso de diuréticos.

Duas semanas depois, ela foi readmitida com piora dos sintomas e hipoxemia. A angioTC mostrou opacidades bilaterais em vidro fosco centrolobulares, linfadenopatia subcarinal e hilar – s 1C e 1D. Foi realizado um teste de caminhada de 6 minutos (TC6M), com distância percorrida de 102 metros e dessaturação de oxigênio acentuada (nadir de 72%). Os TFP mostraram deterioração adicional da capacidade de DLCO (42% do previsto).

Considerando a piora clínica após a terapia vasodilatadora e os achados radiológicos, se reforçou o diagnóstico presuntivo de HAP com envolvimento venoso, o que levou à suspensão do tadalafil. A paciente recebeu alta com ambrisentana e foi encaminhada para transplante pulmonar (TP).

Nos meses seguintes, a paciente apresentou múltiplas reinternações devido ao agravamento dos sintomas. Tentou-se um tratamento experimental com metilprednisolona intravenosa e micofenolato de mofetila, com melhora sintomática de curto prazo. No entanto, foi reinternada novamente 4 meses depois. A paciente permanece em acompanhamento ambulatorial, na classe funcional III da OMS, aguardando transplante hepático.

## Discussão

A HAP com envolvimento venoso/capilar (anteriormente denominada doença veno-oclusiva pulmonar [DVOP]/hemangiomatose capilar) é uma forma rara e grave de HAP, com incidência de 0,1 a 0,5 casos/milhão/ano.^1^ Essa condição compreende um processo fibroproliferativo dos compartimentos venosos e capilares pulmonares, com oclusão de pequenas veias causada por hipertrofia do músculo liso e deposição de colágeno.^2^

O diagnóstico de DVOP é desafiador devido à sua apresentação inespecífica, que muitas vezes mimetiza a HAP. Além disso, a confirmação histológica por biópsia pulmonar é contraindicada devido ao risco significativo de hemorragia. Certas características clínicas e de imagem podem facilitar um diagnóstico presuntivo, incluindo achados típicos na angioTC – opacidades em vidro fosco centrolobulares, linhas septais e linfadenopatia mediastinal, redução acentuada da DLCO nos TFP e nadir de baixa saturação de oxigênio durante o TC6M.^1^ A DVOP se manifesta de duas formas: hereditária, associada a mutações bialélicas autossômicas recessivas no gene EIF2AK4,³ e esporádica, que se apresenta mais tarde na vida e com maior frequência em homens.^1^ Diversos fatores de risco estão associados à DVOP esporádica, incluindo exposição prévia à quimioterapia (particularmente agentes alquilantes)^1,4^ ao tabaco^1^ e a solventes orgânicos como o tricloroetileno.^1,5^

Além disso, o envolvimento venoso foi observado na HAP associada a doenças do tecido conjuntivo (HAP-DTC), particularmente na ES.^6,7^ A DVOP pode coexistir ou surgir como uma complicação da ES, levando à HP por meio de remodelamento venoso pós-capilar distinto. O mecanismo fisiopatológico subjacente da congestão alveolar observada clinicamente — aumento do acúmulo de fluido alveolar após terapia vasodilatadora — é atribuído principalmente à elevação da pressão hidrostática secundária ao remodelamento adverso da vasculatura pós-capilar, envolvendo especificamente as vênulas pulmonares. Mecanicamente, esse remodelamento adverso não é exclusivo da DVOP idiopática, mas é observado consistentemente em várias formas, incluindo aquelas associadas à ES, induzidas por medicamentos ou casos idiopáticos. Nesses casos, os pacientes podem apresentar resposta reduzida ou mesmo piora clínica após o início de terapias vasodilatadoras.^1^

Nossa paciente apresentou características clínicas e laboratoriais indicativas de HAP associada à ES. Sua subsequente deterioração clínica após o início da terapia vasodilatadora, em conjunto com os achados radiológicos, a redução da DLCO e a dessaturação no TC6M, sugeriram fortemente o diagnóstico de doença veno-oclusiva pulmonar.

O tratamento da DVOP envolve medidas de suporte, incluindo oxitocina e diuréticos, além do encaminhamento precoce para transplante hepático. As terapias específicas para HAP têm eficácia e segurança incertas, como demonstrado neste caso, em que a suspensão dos vasodilatadores foi necessária para evitar maior deterioração do quadro clínico.

Apesar disso, o paciente ainda apresentou múltiplas reinternações. Essa evolução clínica está de acordo com a literatura, com rápida progressão dos sintomas e morbidade e mortalidade consideráveis – a maioria dos pacientes morre ou necessita de transplante hepático em até dois anos após o diagnóstico.^1^ Assim, na ausência de terapias eficazes, optou-se pelo encaminhamento precoce para transplante hepático.

Em nosso caso, foi realizado um teste com medicamentos imunossupressores, com melhora dos sintomas e sem reinternações nos quatro meses subsequentes. Embora se acredite que a inflamação contribua para a fisiopatologia da DVOP e da HAP, e alguns relatos tenham sugerido melhora clínica e hemodinâmica com agentes imunossupressores/antiproliferativos, mais pesquisas são necessárias antes que essas abordagens possam ser recomendadas.^1,8,9^

Em 2018, novas variantes patogênicas foram identificadas no gene *ABCC8*, associadas à HAP.^10^ O gene *ABCC8* codifica o receptor de sulfonilureia 1, uma subunidade regulatória do canal de potássio sensível ao ATP. Em uma coorte espanhola de pacientes com HAP, variantes do *ABCC8* foram identificadas em 11 indivíduos, dois dos quais apresentavam HAP-DTC, sendo que um deles manifestava-se com DVOP.^11^ Este paciente era portador de uma variante patogênica do *ABCC8*, que havia sido associada apenas a hiperinsulinismo congênito e diabetes neonatal (ClinVar VCV000035617.15). Até onde sabemos, este é o primeiro caso relatado de HAP-DTC associada a essa variante e o segundo caso envolvendo uma variante patogênica do *ABCC8* em conjunto com evidências clínicas de envolvimento venoso. Infelizmente, a segregação parental não pôde ser investigada devido às limitações geográficas. Estudos adicionais são necessários para esclarecer o papel do *ABCC8* na fisiopatologia da HAP-DTC e da DVOP.

Para concluir, a DVOP representa um subtipo de HP altamente mórbido e com prognóstico reservado. O reconhecimento precoce de suas características é essencial para o encaminhamento oportuno ao transplante hepático, enfatizando que esses pacientes devem ser acompanhados em centros especializados em HP. Relatamos um caso raro e complexo de HAP associada à ES, caracterizada não apenas por envolvimento venoso, mas também por uma variante patogênica previamente não descrita no gene *ABCC8*.

## Data Availability

Os conteúdos subjacentes ao texto da pesquisa estão contidos no manuscrito.
