# Supplementary material for: Hipertensão Arterial Pulmonar com Envolvimento Venoso Associado à Esclerose Sistêmica – uma Nova Variante Patogênica no Gene ABCC8
Source: Arq Bras Cardiol. 2025 Dec 11;122(11):e20250175. [Article in Portuguese] doi: 10.36660/abc.20250175 (PMC12978279; doi:10.36660/abc.20250175)
Supplement: Translation

# Translation: Systemic Sclerosis Associated Pulmonary Arterial Hypertension with Venous Involvement – a Novel ABCC8 Pathogenic Variant

**DOI:** 10.36660/abc.20250175i

**Authors:** Daniel Inácio Cazeiro, João Cravo, Mariana Pereira Silva, João R. Inácio, Oana Moldovan, Catarina Sousa, Catarina Lopes Resende, João Eurico Fonseca, Fausto J. Pinto, Rui Plácido

**Affiliations:** 1 Lisbon Portugal Department of Cardiology, Unidade Local de Saúde de Santa Maria, CAML, CCUL@RISE, Faculty of Medicine, University of Lisbon, Lisbon – Portugal; 2 Lisbon Portugal Department of Rheumatology and Metabolic Bone Diseases, Unidade Local de Saúde de Santa Maria, CAML, Faculty of Medicine, University of Lisbon, Lisbon – Portugal; 3 Lisbon Portugal Department of Radiology, Unidade Local de Saúde de Santa Maria, CAML, CCUL@RISE, Faculty of Medicine, University of Lisbon, Lisbon – Portugal; 4 Lisbon Portugal Department of Medical Genetics, Unidade Local de Saúde de Santa Maria, CAML, CCUL@RISE, Faculty of Medicine, University of Lisbon, Lisbon – Portugal; 5 Lisbon Portugal Center for Disease Mechanisms Research, Faculdade de Medicina da Universidade de Lisboa, Lisbon – Portugal

**Keywords:** Pulmonary Hypertension, Genetics, Therapeutics

A 48-year-old Bissau-Guinean woman with systemic hypertension presented to the emergency department with a 5-month history of worsening fatigue, dyspnea, and peripheral edema. She worked as a cleaner, with no occupational exposures, and had no family history of pulmonary arterial hypertension (PAH). On admission, she was hypoxemic, with bibasilar crackles on auscultation and lower limb edema. Further examination revealed palmar telangiectasias.

Initial investigation revealed hypoxemia and elevated NT-proBNP. The electrocardiogram showed right-axis deviation and signs of right ventricular hypertrophy. Chest computed tomography angiography (CCTA) excluded pulmonary embolism and revealed bilateral ground-glass opacities, dilation of right heart chambers, and pulmonary artery (PA) – Figure 1A – and diffuse esophageal dilation. Transthoracic echocardiogram depicted a D-shaped left ventricle, right heart chamber dilation, severe tricuspid regurgitation, and high echocardiographic probability of pulmonary hypertension (PH).

**Figure 1:** A) Capillaroscopy with active scleroderma pattern - moderate loss of capillary density, giant capillaries (arrows), and microhemorrhages (arrowhead) are evident. B) Chest CT pulmonary angiography demonstrates right atrial and ventricular dilatation, compatible with pulmonary hypertension. C) Follow-up chest CT mediastinal axial images showing bilateral hilar and subcarinal lymphadenopathy. D) Lung axial images depicting diffuse bilateral ground glass opacities in the lower lobes.

The patient was admitted and started on oxygen therapy (OT) and furosemide, with clinical improvement. Further diagnostic work-up of PH was performed by a multidisciplinary team. Capillaroscopy showed giant capillaries and microhemorrhages, consistent with active scleroderma – Figure 1B. Immunology testing detected high titers of antinuclear and anticentromere B antibodies. Pulmonary function tests (PFT) revealed an isolated, severely reduced diffusion capacity for carbon monoxide (DLCO) - 53% of predicted. Serologies for HIV and Hepatitis B/C were negative. Abdominal ultrasound and ventilation-perfusion scans were unremarkable.

Right heart catheterization confirmed precapillary PH, with a negative vasoreactivity test.

A next-generation sequencing (NGS) panel of 16 genes involved in the pathophysiology of PAH identified a pathogenic heterozygous variant in the *ABCC8* gene (NM_000352.6:c.4306C>T; p. Arg1436Te). NGS was performed using the technology Twist Bioscience and a NovaSeq 6000 (Illumina^®^) sequencer. CNV analysis was performed through DRAGEN software (version 07.021.572.3.6.3). No other variants were identified in the panel. A summary of the characteristics and location of the pathogenic variant can be found in Table 1 and Figure 2, respectively.

**Table 1:** Summary of the characteristics of the identified ABCC8 pathogenic variant in the patient

HGVS nomenclature		ABCC8(NM_000352.6):c.4306C>T; p.(Arg1436Ter)
**Chromosomal annotation**		chr11:17395611C>T
**Location**		11p15.1, exon 35, codon 1436
**Mode of inheritance**		Autosomal dominant/recessive
**Zygosity**		Heterozygous (allele frequency 47%)
**Coding impact**		Nonsense
**ACMG Classification**		Pathogenic (PVS1, PM2, PS4)
**Population frequency**		GnomAD Exomes: 0.00000358 GnomAD Genomes: 0.00000657
Pathogenic scores:		
	➢ CADD (phred-scaled)	37
	➢ DANN	0.99
	➢ GERP++	>5.0
	➢ MutationTaster 2025	Deleterious
	➢ NMD Prediction	Positive

Data extracted from ClinVar,^12^ VarSome,^13^ Bohnen et al.^14^ and Lago-Docampo et al.^15^

**Figure 2:** Structural location of ABCC8 variant p. Arg1436Ter. Domain structure of the SUR1 (ABCC8) protein showing the location of the p. Arg1436Ter variant (red line). The variant truncates the second nucleotide-binding domain (NBD2), potentially leading to loss-of-function of the SUR1 subunit.

These findings confirmed the diagnosis of PAH associated with systemic sclerosis (SSc) - limited form. Vasodilator therapy with tadalafil and ambrisentan was initiated. The patient was discharged on the 27th day of admission on diuretic therapy.

Two weeks later, she was readmitted with worsening symptoms and hypoxemia. CCTA showed bilateral centrilobular ground-glass opacities, subcarinal and hilar lymphadenopathy – Figures 1C and 1D. A 6-minute walk test (6MWT) was performed, with a distance of 102 meters and marked oxygen desaturation (nadir 72%). PFTs showed further deterioration of DLCO (42% of predicted).

Given the clinical deterioration following vasodilator therapy and radiological findings, a presumptive diagnosis of PAH with venous involvement was reinforced, leading to discontinuation of tadalafil. The patient was discharged on ambrisentan and was referred for lung transplantation (LT).

In the following months, the patient experienced multiple readmissions due to worsening symptoms. An off-label trial of intravenous methylprednisolone and mycophenolate mofetil was attempted, with short-term symptomatic improvement. However, she was readmitted again 4 months later. The patient remains in the ambulatory setting in WHO functional class III and is awaiting LT.

## Discussion

PAH with venous/capillary involvement (formerly referred to as pulmonary veno-occlusive disease [PVOD] /capillary hemangiomatosis) is a rare, severe form of PH, with an incidence of 0.1-0.5 cases/million/year.^1^ This condition comprises a fibroproliferative process of pulmonary venous and capillary compartments, with small vein occlusion caused by smooth muscle hypertrophy and collagen deposition.^2^

The diagnosis of PVOD is challenging due to its non-specific presentation, often mimicking PAH. Moreover, histological confirmation via lung biopsy is contraindicated due to the significant risk of hemorrhage. Certain clinical and imaging features can facilitate a presumptive diagnosis, which include typical findings on CCTA – centrilobular ground-glass opacities, septal lines, and mediastinal lymphadenopathy, severely reduced DLCO on PFTs, and low oxygen saturation nadir during the 6MWT.^1^ PVOD manifests in two forms: hereditary, which is associated with autosomal recessive biallelic mutations in the *EIF2AK4* gene,^3^ and sporadic, which presents later in life and more frequently in males.^1^ Several risk factors are linked to sporadic PVOD, including prior exposure to chemotherapy (particularly alkylating agents),^1,4^ tobacco,^1^ and organic solvents such as trichloroethylene.^1,5^

Moreover, venous involvement has been observed in PAH associated with connective tissue diseases (PAH-CTD), particularly in SSc.^6,7^ PVOD can coexist or arise as a complication within systemic sclerosis (SSc), leading to pulmonary hypertension via distinct post-capillary venous remodeling. The underlying pathophysiological mechanism of alveolar congestion observed clinically—increased alveolar fluid accumulation following vasodilator therapy—is predominantly attributable to elevated hydrostatic pressure secondary to adverse remodeling of the post-capillary vasculature, specifically involving pulmonary venules. Mechanistically, this adverse remodeling is not unique to idiopathic PVOD but is consistently observed across various forms, including those associated with systemic sclerosis, drug-induced, or idiopathic cases. In these cases, patients may exhibit reduced responsiveness or even clinical worsening after initiation of vasodilator therapies.^1^

Our patient presented clinical and laboratory features indicative of SSc-associated PAH. Her subsequent clinical deterioration after initiation of vasodilator therapy, in conjunction with radiological findings, reduced DLCO, and desaturation on 6MWT, strongly suggested the diagnosis of PVOD.

The management of PVOD involves supportive measures, including OT and diuretics, alongside early referral for LT. PAH-specific therapies have uncertain efficacy and safety, as demonstrated by this case, in which discontinuation of vasodilators was necessary to prevent further decline.

Despite this, the patient still experienced multiple readmissions. This clinical course aligns with the literature, with rapid symptom progression and considerable morbidity and mortality - most patients die or require LT within two years of diagnosis.^1^ Thus, in the absence of efficacious therapies, early referral for LT was pursued.

In our case, a trial of immunosuppressor drugs was performed, with symptomatic improvement and no readmissions over the subsequent four months. Although inflammation is thought to contribute to the pathophysiology of PVOD and PAH, and some reports have suggested clinical and hemodynamic improvement with immunosuppressive/antiproliferative agents, further research is necessary before these approaches can be recommended.^1,8,9^

In 2018, novel pathogenic variants were identified in the *ABCC8* gene, which were associated with PAH.^10^ The *ABCC8* gene encodes sulfonylurea receptor 1, a regulatory subunit of the ATP-sensitive potassium channel. In a Spanish cohort of PAH patients, *ABCC8* variants were identified in 11 individuals, two of whom had PAH-CTD, with one presenting feature of PVOD.^11^ This patient carried a pathogenic *ABCC8* variant, which had only been associated with congenital hyperinsulinism and neonatal diabetes (ClinVar VCV000035617.15). To our knowledge, this is the first reported case of PAH-CTD associated with this variant, and the second case involving an *ABCC8* pathogenic variant in conjunction with clinical evidence of venous involvement. Unfortunately, parental segregation could not be pursued due to geographic limitations. Further studies are warranted to clarify the role of *ABCC8* in the pathophysiology of PAH-CTD and PVOD.

In conclusion, PVOD represents a highly morbid PH subtype with poor prognosis. Early recognition of its characteristic findings is essential for timely referral for LT, emphasizing that these patients should be managed in specialized PH centers. We report a rare, complex case of PAH associated with SSc, not only characterized by venous involvement but also linked to a previously undescribed pathogenic variant in the *ABCC8* gene.

## Data Availability

The underlying content of the research text is contained within the manuscript.
